# Stretchable, Fully Polymeric Electrode Arrays for Peripheral Nerve Stimulation

**DOI:** 10.1002/advs.202004033

**Published:** 2021-02-05

**Authors:** Estelle A. Cuttaz, Christopher A. R. Chapman, Omaer Syed, Josef A. Goding, Rylie A. Green

**Affiliations:** ^1^ Department of Bioengineering Imperial College South Kensington London SW7 2AZ UK

**Keywords:** conductive elastomer, conductive polymer, electrode characterization, flexible bioelectronics, laser manufacturing, peripheral nerve cuff

## Abstract

There is a critical need to transition research level flexible polymer bioelectronics toward the clinic by demonstrating both reliability in fabrication and stable device performance. Conductive elastomers (CEs) are composites of conductive polymers in elastomeric matrices that provide both flexibility and enhanced electrochemical properties compared to conventional metallic electrodes. This work focuses on the development of nerve cuff devices and the assessment of the device functionality at each development stage, from CE material to fully polymeric electrode arrays. Two device types are fabricated by laser machining of a thick and thin CE sheet variant on an insulative polydimethylsiloxane substrate and lamination into tubing to produce pre‐curled cuffs. Device performance and stability following sterilization and mechanical loading are compared to a state‐of‐the‐art stretchable metallic nerve cuff. The CE cuffs are found to be electrically and mechanically stable with improved charge transfer properties compared to the commercial cuff. All devices are applied to an ex vivo whole sciatic nerve and shown to be functional, with the CE cuffs demonstrating superior charge transfer and electrochemical safety in the biological environment.

## Graphical abstract

Conductive elastomers (CEs) are used to fabricate fully polymeric bipolar nerve cuff arrays using laser‐based manufacturing processes. Functionality of the device is demonstrated in vitro and translated to ex vivo whole nerve preparations. It is shown that CE cuffs outperform commercial metal‐based devices, supporting their potential as neural interfacing technologies that address charge injection and mechanical limitations of conventional arrays.

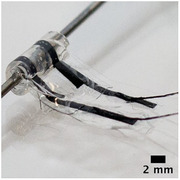

## 1 Introduction

Recently there has been a concerted effort to develop new implantable bioelectronic technologies that can be translated toward clinical application. Although this momentum has been driven by large industry partners in the field of electroceuticals,^[^ ^1^ ^]^ as well as many academic start‐ups,^[^ ^2^ ^]^ there has been little success in replacing the conventional metal‐based approaches used for neural interfacing. This has led to a divide in technological focus between novel research‐based devices with improved performance, and clinically applied devices based on established (or historically regulated) technologies.^[^ ^3^ ^]^ While research‐level novel electrode materials have been used to achieve high fidelity recording and stimulation performance, clinical and industry uptake has been limited, instead focusing on conventional metallic electrodes (primarily platinum or platinum‐iridium) to avoid the risks and regulatory burdens associated with adopting novel biomaterials.^[^ ^3^ ^]^ With an increased interest in using implanted bioelectronics to treat diseases including epilepsy,^[^ ^4^ ^]^ dementias,^[^ ^5^ ^]^ and autoimmune conditions,^[^ ^6^ ^]^ there is a need for devices that can safely deliver high amounts of electrical charge such as those required for neural blocking.^[^ ^7^ ^]^ The low electrochemical charge injection limit of planar metallic electrodes typically used in clinical therapies restricts the ability to safely deliver stimulation at the levels needed for a therapeutic effect in these emerging applications.^[^ ^8^, ^9^ ^]^ Several strategies employing iridium oxide and titanium nitride have been shown to result in improved electrical charge storage capabilities and have been utilized as cardiac pacing electrodes and in lab based high‐density neural interface devices.^[^ ^10^, ^11^ ^]^ Another promising class of biomaterials for neural interfacing are carbon‐based materials including carbon nanotubes and graphene, benefiting from high current injection abilities and high conductivity.^[^ ^12^, ^13^ ^]^ While these technologies provide alternative approaches to addressing the limitations of conventional metal materials, they do not address the challenges with mechanical mismatch, and unlike polymer technologies are not as easily tailored for controlled tissue interactions. As such there remains a critical need for materials with improved electrical and electrochemical performance, coupled with the ability to stretch and flex with the tissues, to be translated from laboratory benches toward clinically relevant devices.

A major characteristic of nerve tissue is that it moves during normal bodily functions, including pulsing with heart beats and displacement with larger musculoskeletal activity such as walking. Many historical nerve‐targeting devices have been fabricated using conventional microfabrication techniques with rigid substrates such as silicon or non‐stretchable polymers such as polyimide. The high stiffness of these substrates results in a mechanical mismatch with the soft and flexible nerve, eliciting an inflammatory tissue response due to continued motion of the nerve around the device. This response can lead to scar tissue encapsulation of the implant and ultimately device failure.^[^ ^14^, ^15^, ^16^ ^]^ Recently, nerve‐targeting devices have seen a shift toward fabrication on soft or compliant substrates such as polydimethylsiloxane (PDMS) or shape memory polymers.^[^ ^17^, ^18^ ^]^

Concurrently, there has been a move away from resource and time intensive photolithography‐based microfabrication toward high throughput laser based micromachining.^[^ ^19^, ^20^, ^21^ ^]^ This has yielded reliable commercial and research‐based devices with improved flexibility. However, flexibility, or rather the ability to stretch and move with tissues, has been enabled primarily through engineering strain relief patterns into thin metal foils and coating of metal electrodes to impart improved charge transfer. This can ameliorate the tissue response, but introduces a new mechanical mismatch, between the stiffer electrode material and soft polymer substrates or coatings. The result has been less than optimal device performance, including reports of electrode failures through fluid ingress or delamination at the site of material property mismatch.^[^ ^22^, ^23^, ^24^, ^25^ ^]^

To realize the next generation of neural interfacing devices within the clinical setting, new functional materials that can improve on both safe charge injection and mechanical compliance, while enabling compatibility with conventional device fabrication must be developed. Designing devices with both soft electrode and substrate materials will dampen the electrode‐tissue mechanical mismatch and reduce the chance of device failure.^[^ ^18^ ^]^ One promising group of materials that meet these requirements are soft and flexible polymer bioelectronics. Conducting polymer (CP) composites in particular has been shown to improve charge transfer while mitigating some of the challenges typically associated with thin film CP coatings such as their poor mechanical durability.^[^ ^26^, ^27^, ^28^, ^29^, ^30^, ^31^, ^32^ ^]^ Both hydrogel and elastomer‐based approaches have been successfully used to stabilize CPs into mechanically robust hybrid materials referred to as conductive hydrogels (CHs) and conductive elastomers (CEs) respectively.^[^ ^26^, ^32^, ^33^, ^34^, ^35^, ^36^, ^37^, ^38^, ^39^, ^40^, ^41^, ^42^ ^]^ CHs have been demonstrated to achieve far superior electrochemical performance when compared to conventional metal electrodes,^[^ ^33^, ^34^ ^]^ however their fabrication has been primarily limited to coating of whole devices or individual electrode sites. This means that although CHs can match the mechanical properties of the nerve tissue and have superior electrochemical properties, they are not processable in a way that can be easily translated to the production of fully polymeric electrode arrays. In terms of processability, CEs are better suited to meet the goal of fully polymeric devices that can be easily and repeatably manufactured using conventional manufacturing techniques. CEs have traditionally had severe limitations due to the tradeoff between electrical performance and mechanical properties.^[^ ^35^, ^43^, ^44^, ^45^ ^]^ However, recent significant improvements in the formulation of highly compliant and conductive CEs have been reported,^[^ ^36^, ^40^, ^41^, ^46^, ^47^ ^]^ leading to preliminary demonstrations of fully polymeric electrodes.^[^ ^41^ ^]^ However, to support the clinical viability of these technologies, there is a need to demonstrate the compatibility of novel CE materials with conventional micromanufacturing techniques to produce entire devices that can be easily handled, sterilized, and remain functionally superior to their metallic counterparts.

This study employed laser microfabrication techniques to produce a functional and mechanically robust CE nerve cuff. The device is developed from initial material production through to a functional electrode array capable of activating nerve fibers. A CE sheet consisting of microfibers of polystyrene sulfonate (PSS) doped poly(3,4‐ethylenedioxythiophene) (PEDOT) dispersed in a polyurethane (PU) elastomer matrix^[^ ^41^ ^]^ was used as the conductive component in the fabrication of a bipolar nerve cuff employing established laser‐based manufacturing practices. By characterizing device performance throughout the fabrication process, benchmarks for this device are established in comparison to state‐of‐the‐art stretchable metallic nerve cuff arrays. Device stability under autoclave sterilization is examined, and the functionality of CE devices is assessed and compared directly to commercial controls using an explanted rat whole sciatic nerve preparation. Devices are shown to be electrically stable under repeated mechanical stretching, showing the mechanical robustness of devices enabled through use of mechanically similar elastomeric components.

## 2 Results and Discussion

A common approach for peripheral nerve cuffs is to have them pre‐formed to specific nerve diameters,^[^ ^48^ ^]^ reducing the need for wrapping and suturing, which is required for more conformal arrays. A poorly fitting nerve cuff can cause undesirable stimulation performance due to excess fluid ingress creating pockets between the nerve and device, or a low seal impedance at the edges, leading to charge leakage and off target effects.^[^ ^49^ ^]^ To facilitate variation in device thickness, CE sheets of varied thickness were fabricated into arrays and performance was assessed in a planar format. Subsequently, two of these thicknesses were laminated within circular tubing to form nerve cuff arrays with a nominal inner diameter of 1 mm.

### 2.1 Electrochemical Characterization of CE of Different Thicknesses

**Figure 1:**
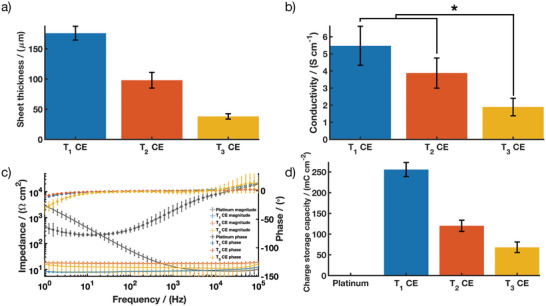
CE sheet performance across three thickness variants (*T*_1_, *T*_2_, and T_3_) showing a) thickness; b) conductivity; c) impedance magnitude and phase angle of EIS; and d) CSC compared to platinum. Results are reported as mean ± standard deviation (*n* = 5). * Significant difference at *p* < 0.05.

CE sheets with 20 wt% PEDOT:PSS were fabricated using methods established in prior studies.^[^ ^41^ ^]^ This formulation was demonstrated to optimally address the trade‐off between mechanical and electrochemical properties required for application in flexible bioelectronics. CE sheets of three different thicknesses were made by varying the volume of pre‐polymer solution used in solvent casting. The measured thickness of the CE variants were *T*_1_ = 176.00 ± 11.40 µm, *T*_2_ = 98.00 ± 13.03 µm and *T*_3_ = 38.00 ± 4.47 µm (**Figure** **1**a). Due to the reduction in material thickness and hence cross‐sectional area, conductivity of the bulk CE was found to decrease from 5.47 ± 1.14 S cm^–1^ to 3.87 ± 0.87 S cm^−1^ and 1.89 ± 0.51 S cm^−1^ for *T*_1_, *T*_2_, and *T*_3_ sheets, respectively (Figure 1b). The charge transfer properties of the bulk CE sheets were characterized by electrochemical impedance spectroscopy (EIS) (Figure 1c), and cyclic voltammetry (CV) in a saline bath. See Figure S1, Supporting Information, for the absolute values. The charge storage capacity (CSC) was calculated using the resulting CV curves and compared to a flat platinum sheet as a control (Figure 1d). The CSC of the CE sheets was reduced as cross‐sectional area was decreased, with CSC values of 255.82 ± 17.09 mC cm^−2^, 120.15 ± 13.55 mC cm^−2^, and 68.28 ± 12.74 mC cm^−2^ for *T*_1_, *T*_2_, and *T*_3_, respectively. These CSC values are comparable to previous reports of thick PEDOT‐based composite films such as conductive hydrogels.^[^ ^50^, ^51^ ^]^ All three thicknesses had similar impedance profiles; at 1 kHz, CE sheets had impedances of 8.71 ± 0.54 Ω cm^2^, 16.69 ± 2.21 Ω cm^2^, and 11.62 ± 5.55 Ω cm^2^ for *T*_1_, *T*_2_, and T_3_ respectively. Impedance behavior was not expected to be highly dependent on CE thickness since it is mostly dependent on the electrode interface and not bulk material properties. The reduction in electrochemical performance seen from the thin CE variants is likely due to the reduction in volume of the CE leading to fewer PEDOT:PSS microfiber network connections being formed. As suggested by percolation theory, a minimum amount of CP is necessary to establish a conductive pathway within an insulative matrix.^[^ ^41^, ^45^, ^52^ ^]^ Although the PEDOT:PSS to polyurethane elastomer ratio was kept constant across all the CE thicknesses tested, reduced sheet thickness eventually led to a reduction in the total amount of PEDOT:PSS to a level not able to form a fully percolated network. The impact of film thickness on the conductivity of solution cast CP‐based polymer blends has been previously investigated. Specifically it was reported that conductivity increased by an order of magnitude when increasing the sheet thickness from 50 to 200 µm.^[^ ^53^ ^]^ It has also been demonstrated that in the case of composites, decreasing sheet thickness resulted in an increased average distance between dispersed conductive particulates.^[^ ^54^ ^]^ This loss in network interconnectivity limits formation of conductive pathways, moving the conductive network away from the percolation threshold, further contributing to a decrease in material conductivity. These results suggest that, in addition to network percolation, the conductivity of conductive composites relies heavily on the overall thickness of the cast material. It should be noted that while the Pt has a high conductivity of 1.01 × 10^5 ^S cm^−1^ far superior to CE conductivities,^[^ ^55^ ^]^ all CE thicknesses had their CSC values at least one to two orders of magnitude larger than that of Pt (1.54 ± 0.20 mC cm^−2^) and their impedance was found to be lower than Pt at low frequencies (<1kHz). This trade‐off between electrical conductivity and charge transfer at a fluid interface is hypothesized to support the development of polymeric bioelectronics with comparable performance to metal electrode technologies while imparting increased electrochemical safe charge injection limits and reduced mechanical failures.

From these thickness studies, *T*_1_ and *T*_2_ were chosen to be fabricated into bipolar electrode arrays. These CE sheets were chosen because they enable further investigation of nerve‐cuff fit without significantly affecting the predicted conductivity of the final device.

### 2.2 Fabrication approach for Fully Polymeric Electrode Arrays

**Figure 2:**
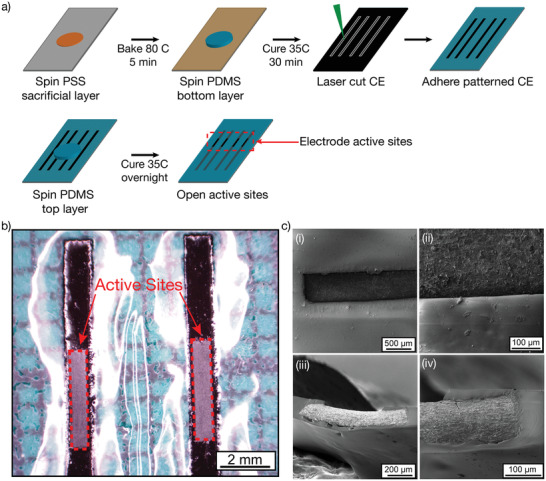
a) Schematic of the nerve cuff device fabrication process. Briefly, a first layer of PDMS was spin‐coated onto a PSS sacrificial layer. CE sheets were laser cut into tracks and were then deposited on the tacky PDMS to ensure proper adhesion between the two layers. A second layer of PDMS was applied to insulate the CE tracks. Finally, the CE active electrodes were exposed to obtain bipolar electrode arrays. b) Stereoscope image of the CE‐based electrode arrays in a flat configuration, showing the electrodes’ active sites. c) SEM image of the electrodes’ active sites i) top‐down view of CE electrode in PDMS insulation, ii) magnified electrode‐insulation interface, iii) cross‐section of CE in PDMS insulation, iv) magnified cross section.

Fully polymeric electrode arrays consisting of CE tracks and active electrodes with PDMS insulation were fabricated using laser‐based processing as shown below (**Figure** **2**a) using the *T*_1_ and *T*_2_ CE sheets. Direct laser micromachining of CE sheets into electrode arrays with active sites of 900 µm in diameter and tracks of 90 µm in width have been demonstrated in pilot studies,^[^ ^41^ ^]^ however the extent of the patterning resolution of CEs has not yet been fully explored. For peripheral nerve cuffs used for whole nerve activation or blocking, a simple bipolar design, comparable to the commercial state‐of‐the‐art was chosen. The bipolar electrode arrays produced from *T*_1_ CE were measured to have an average active electrode site area of 0.0226 ± 0.0017 cm^2^ and a PDMS insulation thickness of 83.13 ± 5.83 µm (Figure 2b). The *T*_2_ CE active electrode sites had an average area of 0.0245 ± 0.0022 cm^2^ and a PDMS insulation thickness of 67.22 ± 6.50 µm. Laser parameters were iteratively tuned to ensure a clean ablative cut of the CE tracks without polymer melt or burn occurring at the edges. SEM images in Figure 2c show the CE electrode sites and cross‐section of the tracks embedded within the PDMS insulation. It should be noted that upon visual inspection, removal of the fully cured PDMS for CE active electrode openings did not result in any residues of PDMS.

### 2.3 Electrochemical Performance of Planar CE Arrays

**Figure 3:**
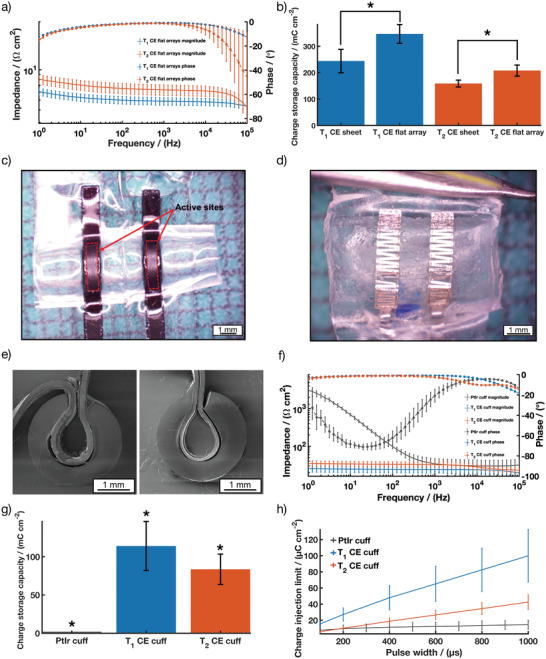
*T*_1_ and *T*_2_ CE flat arrays performance showing a) impedance magnitude and phase angle of EIS and b) CSC plot comparing *T*_1_ and *T*_2_ CE in bulk sheet configuration and in flat array configuration (average across three different batches, *n* = 5 for both *T*_1_ and *T*_2_ CE in bulk sheet; *n* = 14 for *T*_1_ CE flat arrays; *n* = 17 for T_2_ CE flat arrays); Stereoscope images showing a top view of c) a CE cuff array and d) a PtIr cuff array; e) Cross sectional SEM images of *T*_1_ (left) and *T*_2_ (right) CE cuff arrays; *T*_1_ and *T*_2_ CE cuff arrays performance showing; f) impedance magnitude and phase angle of EIS compared to PtIr cuff arrays; g) CSC and h) CIL compared to PtIr cuff arrays. Results are reported as mean ± standard deviation (*N* = 12 for *T*_1_ CE cuff, *N* = 15 for *T*_2_ CE cuff). * Significant difference at *p* < 0.05.

The electrochemical performance of the CE arrays was investigated while flat (planar), prior to their assembly into pre‐curled cuffs (**Figure** **3**). *T*_1_ CE planar bipolar electrode arrays were measured to have a total array thickness of 213.13 ± 15.70 µm, while the thinner CE arrays fabricated using the *T*_2_ CE variant had a total array thickness of 138.89 ± 8.09 µm. The electrochemical properties of the *T*_1_ and *T*_2_ flat CE arrays were characterized by EIS (Figure 3a) and CV (Figure 3b). See Figure S2, Supporting Information, for the absolute values. The CSC for both the array variants were calculated using the CV curves (Figure S3, Supporting Information) and compared to bulk *T*_1_ and *T*_2_ CE sheets (Figure 3b). Characterization of the flat CE arrays showed an impedance of 4.87 ± 0.42 Ω cm^2^ and 6.32 ± 0.76 Ω cm^2^ at 1 kHz for *T*_1_ and *T*_2_ flat CE arrays, respectively as seen in Figure 3a. This correlated to a reduction in impedance of 44% for the *T*_1_ device and 62% for the *T*_2_ device. Similarly, the CSCs of both arrays increased in relation to their bulk CE counterparts to 346.58 ± 35.62 mC cm^−2^ and 207.89 ± 21.00 mC cm^−2^_,_ respectively. Many different factors could have led to the improved electrochemical performance of the flat arrays compared to the bulk CE sheets. Calculations for electrochemical performance were based on measured geometric surface area of the exposed active sites of these CE arrays. However, minor swelling of the CE components^[^ ^7^, ^56^, ^57^ ^]^ may increase the real surface area of the exposed CE, resulting in artificially increased CSC. Additionally, edge effects that increase charge density may result in more efficient charge transfer from the CE array electrode sites compared to bulk CE sheets.^[^ ^58^, ^59^ ^]^ Regardless, it is clearly shown that the CE properties are not negatively impacted by the laser fabrication process, resulting in functional bipolar electrode arrays.

### 2.4 Electrochemical Performance of Bipolar Nerve Cuff Electrode Arrays

To facilitate a fast and easy surgical approach, the planar arrays were laminated within a circular silicone tube (Figure 3c,e). To assess nerve cuff performance, CE nerve cuffs were compared to a commercially available nerve cuff which utilizes bipolar platinum‐iridium (PtIr) electrodes (Figure 3d). The overall device wall thickness (including the pre‐formed tubing) was measured as 744.66 ± 15.43 µm and 653.88 ± 16.03 µm for *T*_1_ and *T*_2_ CE arrays respectively, slightly larger than the device wall thickness of the commercial PtIr cuffs (516.66 ± 11.54 µm). To ensure the functionality of the arrays post‐fabrication, further electrochemical characterization was performed. In addition to measuring the EIS and CSC, the charge injection limit (CIL) of the arrays was compared to PtIr bipolar cuffs. CILs are used to assess electrochemical safety of electrodes by determining the quantity of charge that can be passed through the interfacing material prior to breaching the reduction or oxidation potential at which irreversible chemical reactions can occur. For these and other studies in the literature, this is defined as the potential for water reduction, beyond which irreversible water electrolysis can occur. These reactions are known to produce changes in pH, the evolution of gases and dissolution of metallic electrodes, resulting in damage to the surrounding tissues and electrode degradation.^[^ ^8^, ^60^ ^]^ Figure 3 shows the comparison between the electrochemical performance of both *T*_1_ and *T*_2_ CE cuff arrays and the PtIr cuff array.

The impedance at 1 kHz for the *T*_1_ and *T*_2_ CE cuffs arrays was measured to be 24.07 ± 4.69 Ω cm^2^ and 32.38 ± 5.57 Ω cm^2^, respectively which is similar in magnitude to the PtIr commercial cuff at 34.25 ± 13.01 Ω cm^2^ (Figure 3f). Significant differences can be seen in the phase response of the CE‐based devices when compared to the PtIr device due to the resistive nature of the charge transfer from the CE sheet (Figure 3f). CPs have been shown to be able to combine both electronic and ionic charge transport which leads to more efficient charge transfer when interfacing a biological or wet environment.^[^ ^61^ ^]^ See Figure S4, Supporting Information, for the absolute values. Both the *T*_1_ and *T*_2_ cuff arrays demonstrated statistically significantly higher CSC (113.98 ± 32.06 mC cm^–2^ and 83.56 ± 19.85 mC cm^−2^, respectively) when compared to the PtIr commercial cuff (1.98 ± 0.15 mC cm^−2^) (Figure 3g), resulting in CSCs approximately two orders of magnitude higher than the PtIr cuffs. The resulting CIL of the CE cuffs were significantly improved for all pulse widths tested (Figure 3h). The improved CIL suggests that the CE cuffs will be able to safely deliver more charge to the nerve, without adverse reactions, thus improving the functionality of the devices in both stimulating and blocking modalities.^[^ ^7^, ^16^ ^]^ Consistent electrochemical performance throughout the device fabrication supports that the manufacture process resulted in no or minor residual substances such as PDMS or laser residues, as these would have blocked the CE's surface area for charge transfer, altering the electrochemical properties.

**Figure 4:**
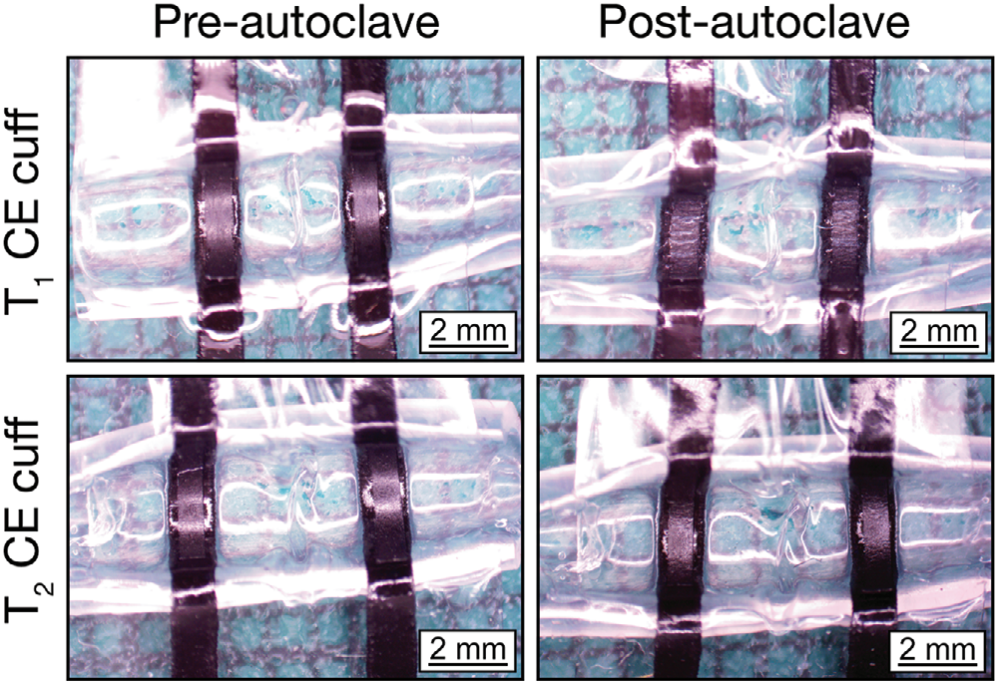
Stereoscopic images showing a top view of *T*_1_ and *T*_2_ CE cuff arrays pre‐and post autoclave.

Stability of the electrodes during the sterilization process is extremely important for nerve cuffs as sterilization is necessary pre‐implantation to ensure minimal risk of infection. Sterilization methods include dry heat sterilization, autoclave, and ethylene oxide sterilization (EtO).^[^ ^62^ ^]^ While EtO is commonly used for bionic devices to prevent damage to electrical hardware components, an alternate clinical sterilization method is autoclaving. Both EtO and autoclaving expose devices to a high‐pressure environment. However, autoclaving is particularly challenging for polymeric materials, probing their thermal stability. International standard specifications for ageing polymers typically involve increased temperature and pressure within a humid environment,^[^ ^63^ ^]^ which can result in material degradation such as mechanical damage or changes in morphology and material structure.^[^ ^64^ ^]^ Therefore autoclaving was used as a rigorous metric to assess a polymer‐based device stability and compatibility with a known sterilization method. Future studies will explore the compatibility of CE devices with EtO sterilization. CE cuffs were analyzed by optical microscopy and electrochemical characterization both pre‐ and post‐sterilization. Stereoscope images of the CE cuffs comparing appearance pre‐ and post‐sterilization showed that both CE cuffs experienced minor morphological changes, with the active sites appearing wrinkled (**Figure** **4**).

**Figure 5:**
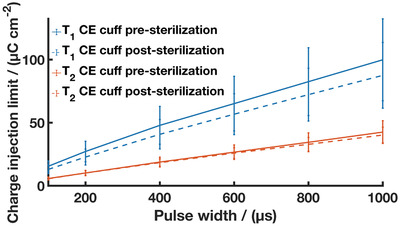
CIL of *T*_1_ (solid blue line + blue dotted line) and *T*_2_ CE cuff arrays pre (solid blue line and solid orange line) and post‐autoclave (dashed blue line and dashed orange line) sterilization. Results are reported as mean ± standard deviation (*N* = 12 for *T*_1_ CE cuffs, *N* = 15 for *T*_2_ CE cuffs).

To assess if these morphological changes altered the device performance, electrochemical studies were undertaken. The electrochemical properties of the CE cuffs pre‐ and post‐autoclave are presented in **Table** **1**. **Figure** **5** shows the change in CIL between the pre‐ and post‐autoclaved CE cuffs for all phase lengths tested.

**Table 1:** Pre‐and post‐autoclave sterilization values for the *T*_1_ and *T*_2_ CE cuffs. Results are reported as mean ± standard deviation (*N* = 12 for *T*_1_ CE cuffs, *N* = 15 for *T*_2_ CE cuffs). A *p*‐value < 0.05 indicates statistical significance (*S*)

Cuff type		Pre‐sterilization	Post‐sterilization	*p*‐value
*T*_1_ CE Cuff	Impedance @ 1 kHz [Ω cm^2^]	24.07 ± 4.69	22.95 ± 5.75	0.22
—	CSC [mC cm^−2^]	113.98 ± 32.06	106.23 ± 31.33	0.10
—	CIL @ 200 µs [µC cm^−2^]	27.16 ± 8.10	22.74 ± 6.30	0.002 (*S*)
*T*_2_ CE Cuff	Impedance @ 1 kHz [Ω cm^2^]	32.38 ± 5.57	36.41 ± 6.91	0.001(*S*)
—	CSC [mC cm^−2^]	83.56 ± 19.85	68.82 ± 13.46	<0.001 (*S*)
	CIL @ 200 µs [µC cm^−2^]	10.12 ± 2.09	10.11 ± 1.80	0.96

Despite morphological changes observed following sterilization, there was minimal change in performance resulting from autoclave treatment of the CE cuffs. The thicker *T*_1_ device saw statistically non‐significant changes in impedance and CSC, and a small decrease in CIL suggesting a negligible impact on the CE electrodes. The thinner *T*_2_ device saw an increase in impedance with a small drop in CSC, but statistically non‐significant change to the CIL. These findings demonstrate autoclave stability for the CE devices and are in line with previous works studying autoclave stability of PEDOT‐based materials. Minimal loss of charge transfer activity including increased impedance and reduced CSC have been reported, with potential mechanisms of change being loss of unbound dopant or polymer chain rearrangement.^[^ ^65^, ^66^, ^67^ ^]^ Importantly, these studies demonstrate the mechanical robustness of CE devices, showing an improvement when compared to CP and CH‐coated metal devices which are prone to cracking and delamination.^[^ ^7^, ^22^, ^68^ ^]^ This further supports the use of CEs as a standalone conductive component.

### 2.5 Nerve Cuff Functionality

**Figure 6:**
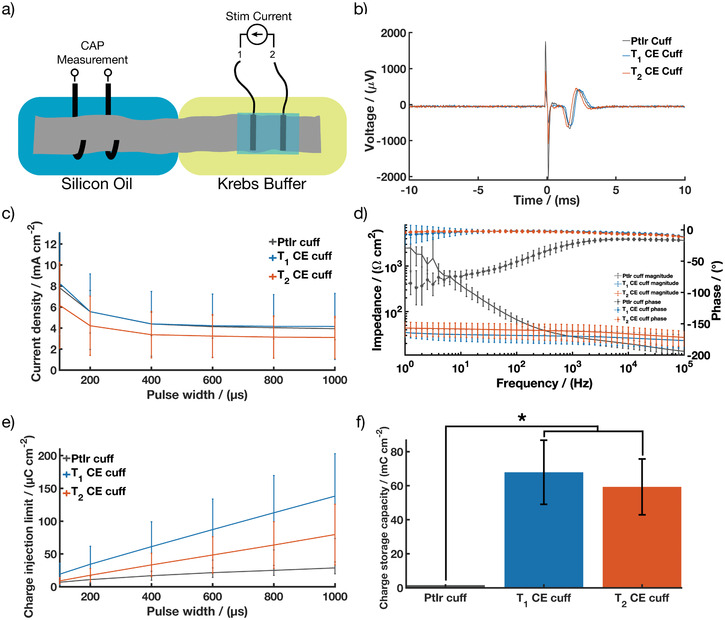
T_1_ and T_2_ CE cuff arrays performance during ex vivo showing; a) Schematic of the ex vivo set up; b) Representative compound action potentials recorded during ex vivo following stimulation by PtIr cuff arrays, *T*_1_ and *T*_2_ CE cuff arrays; c) A‐fiber activation threshold compared to PtIr cuff arrays; d) impedance magnitude and phase angle of EIS compared to PtIr cuff arrays; e) CIL and f) CSC compared to PtIr cuff arrays. Results are reported as mean ± standard deviation (*N* = 11 for *T*_1_ CE cuff, *N* = 15 for *T*_2_ CE cuff). * Significant difference at *p* < 0.05.

An ex vivo rat sciatic nerve preparation was used to evaluate the functional performance of CE electrode arrays (**Figure** **6**a). Ex vivo testing provides a robust method for evaluation of device functionality when tested on their intended anatomical site, herein explanted nerves, as well as helping device design process to optimize key design parameters such as electrode array geometry or device fit to the target site anatomy. The A fiber activation thresholds, contact impedance, CIL, and CSC of the CE cuffs were compared to an equivalent commercial cuff with PtIr electrodes. Figure 6b shows representative electrical activity recorded during ex vivo, showing first stimulation artefact followed by compound action potential of the rat sciatic nerve. Stimulation using a monophasic current pulse of 200 µs duration by the PtIr cuff and the two CE variant cuffs resulted in a similar nerve response, indicative of a successful stimulation and consistent with previous work conducted on the same ex vivo set up.^[^ ^69^ ^]^ Both CE cuff variants were able to stimulate A fiber activity at a current density similar to that of the commercial PtIr cuff (Figure 6c), suggesting that the fit of the cuff on the nerve was similar for all devices. The cuff seal is mainly impacted by the fit of the cuff around the nerve. Since all devices demonstrated similar fit around the nerve, it is not expected that a difference in stimulus dissipation outside the cuffs occurred. EIS spectra of the cuffs demonstrated similar contact impedance for both *T*_1_ and *T*_2_ CE devices (Figure 6d). See Figure S5, Supporting Information, for the absolute values. When in contact with the nerve, both *T*_1_ and *T*_2_ CE cuffs significantly outperformed the PtIr commercial cuffs on the basis of charge transfer characteristics. A statistically significant increase of 214% in CIL at 200 µs was shown for the *T*_1_ cuff and a statistically significant increase of 60% for *T*_2_ cuff, in comparison to the PtIr cuff (Figure 6e). Similarly, there was an approximately 60‐fold increase in CSC when comparing the CE cuffs to the PtIr cuff (Figure 6f). These CSC values are higher than literature values for CP coated peripheral nerve electrodes which have been reported across the range 35–48 mC cm^−2^.^[^ ^40^, ^70^ ^]^ Recently, CH‐coated metallic cuff nerve cuff arrays were tested acutely on rat sciatic nerve and showed significant improvement in electrochemical performance in vivo compared to uncoated metallic cuffs, resulting in a CSC value of 160 mC cm^−2^ and a CIL at 100 µs of 130 µC cm^−2^. While these were higher than that of the CE cuff devices, the coatings were found to be relatively fragile and highly reliant on the mechanical stability of the underlying metal cuff device.^[^ ^51^ ^]^ While CH‐coated cuff arrays provide a softer interface to enhance nerve contact with less trauma, CE devices have the benefit of being entirely made from soft and stretchable components, able to move with the dynamic musculoskeletal and nervous system. Figure S6, Supporting Information shows representative voltage transients obtained during ex vivo while activating A fibers. The monophasic stimulation for PtIr cuffs, *T*_1_ CE cuffs and *T*_2_ CE cuffs were captured at a pulse width of 300 µs and a current amplitude of 500 µA. Both the PtIr cuff and the *T*_1_ CE cuff have a similar voltage at the end of phase, while the *T*_2_ CE cuff shows a significantly larger voltage, indicative of a larger access voltage. Both CE cuffs display voltage transient waveforms with a square shape, typical of CP‐based materials and suggesting a resistive charge transfer behaviour, as previously reported.^[^ ^41^, ^71^ ^]^

CEs provide flexibility in device design and manufacturing, which may support clinical translation across different applications targeting alternate nerves or other electroexcitable tissues. It should be noted that while the *T*_2_ CE cuff underperformed compared to the thicker *T*_1_ CE cuffs, this thinner CE cuff variant still exhibited enhanced electrochemical performance on the nerve compared to PtIr cuff. This suggests that thin CE electrodes could be a feasible way to maintain improved performance and move away from needing specific device diameters for different nerve sizes. CE compatibility with laser machining processes enables the design and fabrication of thin (<150 µm), conformal and potentially high density electrode array devices. Thin conformal CE devices would enable a “one size fits all” approach for nerve cuffs and expand opportunities to other neural interfaces and biosensing such as electrocorticography grids and wearable sensors.

To understand the mechanical robustness of devices and ensure reliability, the preservation of performance was assessed following removal from the nerves. Table S1 and Figure S7, Supporting Information, show that after nerve stimulation, the CE cuffs experienced minor changes in electrochemical performance. Pre and post ex vivo values for *T*_1_ CE cuff channels showed a decrease in electrochemical performance, reflected by a statistically non‐significant increase in impedance at 1 kHz from 23.02 ± 5.72 Ω cm^2^ to 25.27 ± 3.00 Ω cm^2^, a statistically significant decrease in CSC from 105.05 ± 32.28 mC cm^−2^ to 89.07 ± 13.68 mC cm^−2^ and a statistically significant reduction in CIL at 200 µs from 22.61 ± 6.26 µC cm^−2^ to 16.13 ± 2.73 µC cm^−2^. The thinner *T*_2_ CE device had statistically significant changes in electrochemical performance, with a small decrease in impedance at 1 kHz from 36.41 ± 6.91 Ω cm^2^ to 32.29 ± 10.02 Ω cm^2^, an increase in CSC from 68.82 ± 13.46 mC cm^−2^ to 79.13 ± 20.99 mC cm^−2^, and a higher CIL at 200 µs going from 10.11 ± 1.80 µC cm^–2^ to 11.70 ± 3.32 µC cm^–2^. The PtIr cuffs maintained stable electrochemical performance, with statistically non‐significant changes in impedance at 1kHz which decreased from 27.28 ± 11.63 Ω cm^2^ to 22.73 ± 2.35 Ω cm^2^ and in CSC, which increased from 2.29 ± 0.35 mC cm^–2^ to 2.51 ± 0.55 mC cm^–2^, while the CIL was significantly reduced from 10.65 ± 1.81 µC cm^–2^ to 9.69 ± 2.64 µC cm^–2^. These results are indicative of a minor impact from the ex vivo experiment and handling of the device. The variability in electrochemical performance observed for the CE cuffs might be due to slight swelling of the CEs, as evidenced during visual inspection of the cuffs following ex vivo testing where the cuffs are immersed in Krebs buffer for a prolonged amount of time. Additionally, these changes in electrochemical properties are in line with prior studies where CPs show an initial change in properties that can be attributed to loss of dopant or CP chain rearrangement. These mechanisms have been observed in studies on CP coated electrodes through accelerated ageing and high frequency electrical stimulation of the materials.^[^ ^65^, ^72^ ^]^ A drop in electrochemical properties was observed but shown to plateau to stable longer term performance. It is important to note that despite these minor changes in performance, the CE cuffs remain functional following the *ex vivo* studies, maintaining their electrochemical benefit over PtIr devices. In addition to the electrochemical performance, the CE cuffs were imaged with a stereoscope after explantation and it was confirmed that no mechanical failures occurred from handling or use (Figure S8, Supporting Information).

This ex vivo study demonstrates that CE devices preserve their superior electrochemical properties throughout the process of fixation onto a nerve, stimulation, and removal. This improvement in electrochemical performance is especially relevant for addressing key issues in the current trend of electroceuticals where the blocking and stimulation of higher threshold fibers (C fibers) in the parasympathetic and sympathetic nervous system is required.^[^ ^73^, ^74^ ^]^ Due to the high current threshold of these fibers, many metal‐based devices are unable to safely deliver the charge necessary. The use of CE‐based devices has the potential to improve the long‐term safety of the electroceutical devices as well as lead to further advancements in the resolution of these devices.

### 2.6 CE Cuff Behavior under Cyclic Tensile Testing

Both components of the CE cuff have been shown to display elastomeric‐like properties, with a Young's modulus of 1.32–2.97 MPa for the PDMS^[^ ^75^ ^]^ and 38.31 MPa for the CE sheets,^[^ ^41^ ^]^ making the final device mechanically compliant and soft, with a substantially lower Young's modulus than traditional Pt metals (168 GPa^[^ ^76^ ^]^). Having tissue‐contacting materials with properties closer to tissues mechanics contributes to minimizing the mechanical mismatch at the tissue‐device interface. However, in the context of device development, additional investigation on the mechanical behaviour of the end‐device is critical. While the individual components of the CE cuff have been shown to be stretchable and the CE cuff devices were not damaged during experiments, it is important to assess the ability of the cuffs to withstand mechanical stresses and strains induced during handling and implantation. Therefore, a cyclic tensile testing study was conducted. CE cuffs and PtIr commercial cuff arrays were stretched to an opening of 3mm (mimicking an approximate surgical placement around a 1mm nerve) for 100 cycles. It is noted that this is beyond that which would be expected for any device but was used to reflect the need for substantial handling and movement during manufacture, quality control testing, and shipping.

**Figure 7:**
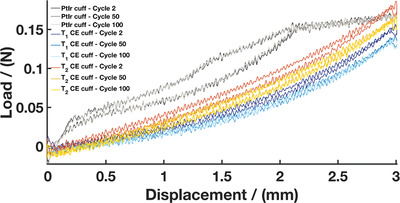
Representative load–displacement curves during cyclic tensile testing for the *T*_1_ CE cuff, *T*_2_ CE cuff, and PtIr cuff (*N* = 5 for *T*_1_ CE cuffs, *N* = 5 for T_2_ CE cuffs, *N* = 2 for PtIr cuffs)

Representative cyclic load‐displacement curves are depicted in **Figure** **7**. Both *T*_1_ and *T*_2_ CE cuffs exhibited a similar viscoelastic behavior, typical of elastomer‐based materials.^[^ ^77^, ^78^ ^]^ Loading and unloading cycles led to a small amount of hysteresis in the load‐displacement curves, corresponding to the energy dissipated during cycling. This behavior is attributed to internal structural reorganization of the polymeric networks of viscoelastic materials such as silicone. The hysteresis tended to stabilize after the first few cycles, suggesting that internal rearrangement happening during initial deformation was maintained for the rest of the stretching cycles and that no other changes occurred. These findings are in agreement with literature investigating cyclic stretching of elastomer based materials.^[^ ^45^, ^78^ ^]^ The PtIr cuffs displayed similar viscoelastic behavior, but experienced higher loads compared to the CE devices. This suggests that the polymeric devices require less force to be fully opened than the PtIr‐based devices, which may prove beneficial to implant procedures where accidental crush of nerves can occur.

The influence of cyclic tensile testing on the device performance was monitored by optical microscopy and electrochemical characterization both before and after mechanical testing. Stereoscope images of the CE cuffs pre‐ and post‐ mechanical testing demonstrated that both *T*_1_ and *T*_2_ CE cuffs show no visible signs of damage (Figure S9, Supporting Information). The CE active sites and tracks did not suffer from fracture and no evident delamination or insulation breakdown was observed. These observations can be attributed to a reduction in the mechanical mismatch between the CE components and the silicone‐based substrate layers. It was observed that the laminated flat array of one CE cuff was being pulled away from the silicone tubing when stretched. This is likely due to limited spreading of the silicone glue inside the pre‐curled tubing. A non‐uniform glue layer induces a stress concentration point in the device, but this can be easily addressed by more even spreading. No major changes were seen on the PtIr cuffs following cyclic tensile testing, but some movement of the PtIr serpentine electrode tracks was observed. This is indicative of the mechanical mismatch and poor adhesion between the metal electrode and the silicone‐based insulation. Ultimately, this mismatch can lead to fluid ingress around the electrodes and bonding sites.^[^ ^7^ ^]^ Ensuring adequate adhesion of the conductive components to the insulation layer is an important factor to prevent delamination and is one of the most common failure modes in implanted devices, resulting in charge leakage and channel cross talk.^[^ ^22^, ^24^, ^79^ ^]^ While optical microscopy confirmed that repeated mechanical stretching did not alter the CE cuff integrity, electrochemical studies were performed to correlate these observations. The electrochemical properties of the CE cuffs and PtIr cuffs pre‐ and post‐cyclic tensile testing are shown in Table S2, Supporting Information. Overall, there was minimal change in performance following cyclic tensile testing of the CE cuffs. The *T*_1_ CE cuff had statistically non‐significant changes in electrochemical performance, including a slight decrease in impedance at 1kHz from 27.61 ± 5.14 Ω cm^2^ to 25.53 ± 4.67 Ω cm^2^, an increase in CSC from 86.37 ± 15.02 mC cm^–2^ to 91.59 ± 19.83 mC cm^–2^ and a negligible change in CIL at 200 µs from 17.02 ± 3.32 µC cm^–2^ to 17.30 ± 3.17 µC cm^–2^. The thinner *T*_2_ CE device appeared to be more effected by repeated stretching but showed improved electrochemical performance. The *T*_2_ CE cuff recorded a statistically significant drop in impedance at 1 kHz from 40.84 ± 10.43 Ω cm^2^ to 33.89 ± 11.10 Ω cm^2^, a statistically significant increase in CSC from 63.71 ± 17.13 mC cm^–2^ to 76.01 ± 18.92 mC cm^–2^ and a small increase in CIL from 10.69 ± 1.43 µC cm^–2^ to 13.76 ± 4.42 µC cm^–2^. It is likely that the repeated stretching of the CE component enabled some degree of fiber rearrangement, improving communication of the PEDOT:PSS component. The PtIr cuff device saw small but non‐significant changes, including a small decrease in impedance at 1 kHz from 22.35 ±1.57 to 20.02 ± 3.86 Ω cm^2^, an increase in CSC from 2.89 ± 0.25 mC cm^–2^ to 3.26 ± 0.91 mC cm^–2^ and an increase in CIL of 11.05 ± 1.60 µC cm^–2^ to 12.66 ± 0.96 µC cm^–2^. Overall, cyclic tensile testing did not alter physical integrity of the CE cuffs. Both CE and PtIr cuffs were found to remain functional, demonstrating the stretchability and mechanical robustness of these devices. The ability to undergo mechanical deformation without significant damage is critical for implanted devices, enabling easier handling and reducing the risk of device failure throughout usage.

Throughout this study, the CE technology was compared to commercially available metallic cuffs considered state‐of‐the‐art for recording and stimulation of peripheral nerves, combining PtIr electrodes with a flexible serpentine pattern, designed to impart increased electrode surface area while having mechanical compliance. The CE cuffs were shown to meet the same performance requirements as the PtIr cuff in terms of functionality and mechanical robustness, with the benefit of enhanced electrochemical properties. Another important consideration is the inherent flexibility of the CE material, reducing the chance of device failure that can arise from mechanical mismatch between electrode material and soft silicone‐based insulation, while imposing no design constraint on the device. As the CE stretches with the insulation, there is no need for complex strain relief patterns such as serpentine structures to impart stretchability to the device, which precludes the ability to have densely packed electrode configuration. Therefore, this intrinsic stretchability enables modularity in electrode design and facilitates the design of stretchable, high density electrode arrays, coupled with rapid and straightforward manufacturing using laser‐based processes. These characteristics support the further development of CE devices into clinically applied electrode arrays that can address the charge transfer limitations and failure mechanisms of current technologies.

Appropriate biological performance, including cytocompatibility, stability within the biological environment, and chronic performance, is fundamental for devices intended to be used for implantable bioelectronics. In vitro cytotoxicity studies have shown that the CE sheets were cytocompatible.^[^ ^41^ ^]^ Additionally, both components of the CE composites have been found to be stable in prior literature. In the case of the CP component, accelerated electrical ageing studies have shown that PEDOT coated electrode arrays demonstrate stable electrochemical and electrical performance following 2.8 billion cycles of clinically relevant biphasic stimulation.^[^ ^72^ ^]^ Accelerated chemical ageing has shown that PEDOT based materials retain electrical performance and chemical stability over 2 years of simulated body conditions.^[^ ^80^ ^]^ In a different study, PEDOT:PSS coated electrodes were found to have stable performance in cell culture conditions for at least four months with minimal change in morphology.^[^ ^81^ ^]^ Finally, PEDOT‐based CHs on cochlear implants have been reported to retain their electrical performance after 2 billion stimulations in artificial perilymph, mimicking 2 years of implantation.^[^ ^71^ ^]^ However, one consideration for material stability is the possible release of leachables, especially from the PEDOT:PSS complex, where PSS chains are used in excess to PEDOT doping requirements to facilitate solubility and dispersion. Indeed, it has been reported that loosely bound PSS within the PEDOT:PSS complex can be released into the surrounding aqueous environment^[^ ^82^ ^]^ causing cytotoxicity and cell growth inhibition due to the acidic nature of PSS.^[^ ^83^ ^]^ Several approaches have been developed to address this limitation, and the most simplistic is to remove the PSS by washing or soaking in an aqueous sink.^[^ ^81^, ^84^, ^85^ ^]^ Alternately, using stabilizers or encapsulating the PEDOT:PSS in a bulk matrix can restrict PSS movement and prevent leaching.^[^ ^82^, ^83^ ^]^ In the case of the CE technology, the PEDOT:PSS is encapsulated in an elastomeric matrix, being medical grade polyurethane elastomer Pellethane. This was specifically chosen as it has been widely used in medical implants such as cardiac pacemakers owing to its flexibility and biocompatibility.^[^ ^86^ ^]^ However, it has been reported that this material could be prone to slow hydrolytic degradation in vivo.^[^ ^87^ ^]^ To evaluate the impact of these several factors on the long term performance of the CEs, future studies will seek to evaluate the stability of CE composites under accelerated ageing in biologically relevant milieu. It should however be noted that upon device implantation, the exposed CE will be minimal, being only the electrode interface, which will oppose the fatty tissues around the peripheral nerve sheath. Importantly, the adjacent tissues will primarily interface with the PDMS insulation, which has a long history of safe use as an implant material in biomedical applications such as cochlear implants, blood pumps, pacemaker lead insulators, and catheters, owing to its mechanical robustness, durability, thermal and oxidative stability, cytocompatibility, and bioinertness.^[^ ^88^ ^]^ Consequently, the CE composites herein are considered to be low risk for degradation and toxic leachants due to encapsulation within the PDMS insulation, which constrains and limits the ability for any mobile components to reach the surrounding biological tissues. Future work will study the performance of CE cuffs in a chronic in vivo setting, to investigate the long‐term tissue response and establish the device efficacy, biocompatibility and stability under active stimulation and recording, compared to conventional PtIr cuffs.

## 3 Conclusion

The translation of high‐performance materials into mechanically robust implanted devices has remained a significant barrier toward the widespread translation of novel electroactive materials into clinical applications. This is especially true for flexible and stretchable polymer‐based materials that suffer from interfacial failures when used in conjunction with traditional electronics. CEs are promising materials due to their mechanical stability and ease of processability. These materials were shown to be compatible with conventional laser fabrication techniques used to make commercial devices. Bipolar CE nerve cuff devices were fabricated from CE sheets and found to have stable performance throughout manufacturing, general handling, sterilization, cyclic tensile testing and model implantation on an ex vivo whole nerve. Ex vivo fibre activation threshold analysis demonstrated that fully polymeric CE devices were functionally equivalent to commercial PtIr devices, with significantly improved CIL. This increase in CIL will facilitate miniaturisation of electrodes, enabling the development of high‐density microelectrode arrays which are of interest within peripheral nerve stimulation strategies, such as spatial current steering for selective stimulation, as well as broader bioelectronic applications which require high density arrays. Ultimately, CEs present a promising material platform on which to build the next generation of nerve stimulation electrodes.

## 4 Experimental Section

##### Fabrication of Conductive Elastomers (CE)

CE sheets were fabricated as per previous publication.^[^ ^41^ ^]^ Briefly, PU (Pellethane 2363–80AE Polyurethane Elastomer, Ether Based) was dissolved in dimethylacetamide (DMAC) (5 wt%, w/v) by mixing at 60 °C for 24 h. Lithium perchlorate (LiClO_4_) (0.16 wt%, w/v) was added to the mixture followed by an additional stirring for 10 h. Subsequently, poly(3,4‐ethylenedioxythiophene):polystyrene sulfonate (PEDOT:PSS) microfibers (dry re‐dispersible pellets, Sigma–Aldrich) were dispersed at a PEDOT loading of 20 wt% in the DMAC‐polyurethane solution and mixed under mechanical stirring for 72 h at a temperature of 60 °C. The resulting solution was solvent cast into glass plates and dried in a vacuum oven at 65 °C for 36 h to produce thin films of CE.

##### Fabrication of Bipolar Nerve Cuff Electrode Arrays

First, a sacrificial release layer of 20 wt% polystyrenesulfonic acid (PSSA) was spin‐coated onto 52 × 76 mm glass slides (Marienfeld, Germany) at 400 rpm for 10 s and 3000 rpm for 45 s. The release layer was then cured on a hot plate at 80 °C for 5 min. Next, polydimethylsiloxane (PDMS) (Sylgard 184, Dow Corning Corporation, Michigan, USA) was prepared at a weight ratio of 10:1 (base:curing agent), degassed, and spin‐coated onto the PSSA layer at 400 rpm for 10 s and 2000 rpm for 45 s. The PDMS layer was subsequently cured for 1 h at 34 °C on a hot plate until tacky. CE sheets were laser cut (VersaLASER 2.30DT CO_2_ Laser system, Universal Laser Systems) into 20 mm x 1.2 mm tracks using 0.5% power, at 250 PPI and at a maximum cutting speed of 0.254 m s^–1^. The CE tracks were then applied onto the tacky PDMS layer to ensure sufficient bonding between the two materials. A 3 mm spacing was achieved between CE tracks to obtain a bipolar electrode array configuration. Another PDMS layer was subsequently spin‐coated as an insulation layer using the same parameters as used previously. Following overnight curing at 34 °C, electrode active sites and contact pads with dimensions 4 mm x 0.8 mm were exposed with a scalpel and excess PDMS was removed manually. Exposure of electrode sites and contact pads can be performed by laser ablation using a protocol previously reported for CE electrode arrays.^[^ ^41^ ^]^ In this work, removal of the silicone insulating layer was performed manually due to an ongoing technical problem with the available laser system. However it should be noted that laser ablation of PDMS insulation has been widely reported in literature for electrode array manufacture. The outlines of the arrays were defined with a scalpel to result in a final device dimension of 30 mm x 10 mm. The arrays were then immersed into deionized water before being gently peeled off from the glass substrate using tweezers. Finally, the arrays were connected to platinum wires (125 µm diameter, polytetrafluoroethylene insulated, Advent Research Materials, UK), insulated with silicone (NuSil MED4‐4220, Polymer Systems, UK), and assembled into a cuff device by using PDMS to glue the arrays into silicone tubing of 10 mm in length, 1.5 mm in internal diameter, and 0.5 mm in wall thickness (T1.5×0.5ST60, Polymax Ltd, Hampshire, UK). The finished arrays were then cured overnight on a hot plate at 34 °C to ensure mechanical durability before use.

##### Device Imaging

Images were acquired with a scanning electron microscope (Lyra3, Tescan, Czech Republic) at an acceleration voltage of 1 kV.

##### Conductivity Measurements

A two‐probe set up was utilized to measure conductivity of CE sheets. Samples were clamped at each end using platinum sheets to connect them to a potentiostat (Multi Autolab/M101, Eco Chemie, Netherlands). Sample resistivity was acquired by extracting the impedance output at 1 kHz from EIS measurements. The conductivity (*σ*) was derived using the following Equation (1):

(1) $$\sigma =L/\left(R\times A\right)$$

wherein *L* = length between the clamps, *R* = recorded impedance, *A* = cross‐sectional area of the sample. Results are reported as means over three samples.

##### In Vitro Electrochemical Characterization

Electrochemical characterization was assessed by performing electrochemical impedance spectroscopy (EIS) and cyclic voltammetry (CV) using an Autolab potentiostat (Multi Autolab/M101, Eco Chemie, Netherlands). All measurements were conducted using a three electrode set up with a platinum wire counter electrode and an isolated silver/silver chloride (Ag/AgCl) reference electrode immersed in Dulbecco's phosphate buffered saline (Sigma). EIS was measured using a 10 mV sinusoidal voltage at frequencies ranging from 0.1 to 10 kHz. Bode plots of the impedance magnitude spectra and phase shift spectra were analyzed. CV curves were obtained by sweeping the voltage between −0.6 to 0.8 V at a scan rate of 150 mV s^–1^ for a total of 11 cycles and measuring the resulting current. Charge storage capacity (CSC) values were computed by integrating the current response of the last CV cycle with respect to time.

##### In Vitro Electrochemical Charge Injection Limit

Charge injection limits (CIL) were determined by analyzing the voltage transient response to a current‐controlled biphasic stimulation pulse. The experiment was conducted in the same three‐electrode set up used for EIS and CV. The CIL was defined as the charge necessary to polarize the electrode–electrolyte interface to the water reduction potential (*E*_mc_ = −0.6 V), obtained by subtracting the access voltage (*V*_a_) at the onset of the pulse from the maximum negative voltage transients. Charge balanced cathodic‐first current pulses with a 20 µs interphase delay were delivered at 100, 200, 400, 600, 800, and 1000 µs pulse widths using a stimulator (STG4002‐16mA, Multichannel Systems, Reutlingen, Germany). Current amplitudes were incrementally increased until the output voltage profile measured with an oscilloscope (TBS2000, Tektronix) reached an *E*_mc_ of −0.6 V.

##### Autoclave Sterilization

Sterilization was achieved by placing the cuff devices into sealed pouches and exposing them to high pressure steam at 121 °C using an autoclave (Model MLS‐3751, SANYO, Japan). To assess the impact of the sterilization process, the electrochemical properties were remeasured following autoclaving. Additionally, visual inspection of the cuffs was performed before and after sterilization using a stereoscope to assess any morphological changes (OZL 963 Stereo Microscope, Kern).

##### Ex Vivo Characterization

Detailed experimental procedure can be found in the Supporting Information. In brief, female Sprague–Dawley with a weight range of 200–300g (Charles River, UK) were humanely euthanized under anesthesia and both sciatic nerves were dissected. The nerve was then placed in the nerve chamber (Figure S10, Supporting Information) and fed through to both chambers and fixed in place using insect pins. The nerve was maintained in a modified Krebs buffer at a temperature of 37 °C ,oxygenated using a gas dispersion tube and carbogen gas (95% Oxygen / 5% Carbon Dioxide) (BOC, UK) which was fed into the nerve chamber using a gravity siphon at a flow rate of 8.45 mL min^–1^. The modified Krebs buffer consisted of: NaCl_2_ (113 mm), KCl (4.8 mm), CaCl_2_ (2H_2_O) (2.5 mm), KH_2_PO_4_ (1.2 mm), MgSO_4_ (1.2 mm), NaHCo_3_ (25 mm), and Dextrose (5.55 mm) (Sigma). The buffer was then removed from the oil chamber and filled with mineral oil (Sigma). The drainage was carried out using a peristaltic pump (Pump P‐1, Pharmacia Biotech, Sweden) and this was fed back into the buffer reservoir. CE‐based cuffs and metal‐based control cuffs (PtIr contacts, internal diameter 900 µm, AirRay research Micro Cuff Tunnel, CorTec Freiburg, Germany) were placed around the nerve using forceps. Ex vivo performance was characterized by recording A fiber activation thresholds, EIS, CV, and CIL. All measurements were conducted using one channel of the bipolar cuff as the working electrode, the other channel as the reference electrode and a distant platinum sheet as the counter electrode. First the cuffs were tested to confirm A‐fiber stimulation of the nerve. A monophasic test stimulation was carried out with a pulse width of 300 µs and a current amplitude of 500 µA, with five repetitions to ascertain the health of the nerve. Stimulation was delivered through a custom‐built stimulator. Signals were amplified 100 times with an amplifier (Model SR560, Stanford Research Systems, USA) and 50Hz noise was removed using a Humbug (Digitimer, USA). A maximum nerve response was established using the oscilloscope (in mV) (Wavesurfer 434, LeCroy, USA) at the test stimulation level of pulse width of 300 µs and a current amplitude of 500 µA. Stimulation which elicited a clear compound action potential (CAP) which was 50% and above of the negative peak‐to‐baseline amplitude was taken as a successful nerve stimulation. This was measured at incrementally increased pulse width value from 100 to 1000 µs. EIS and CV were conducted using parameters previously described in the in vitro baseline characterization (PGSTAT204, Eco Chemie, Netherlands). The charge injection limit was then measured using a biphasic stimulation and using equipment arrangement up with the addition of an active differential probe (AP 034, LeCroy, USA). The charge–injection limit was defined as the quantity of charge necessary to polarize the electrode interface to an *E*_mc_ of −0.8 V. A range of stimulation at different pulse widths from 100 to 1000 µs were delivered in a randomized fashion to record the CIL.

##### Mechanical Behavior

Cyclic uniaxial tensile tests were conducted using an Instron 5543 tensile testing machine (Instron Inc., Canton, MA) equipped with 100N load cell. The cuffs were clamped at the flaps to the tensile grips and were stretched for 100 cycles at a constant crosshead speed of 30 mm min^–1^. The maximum crosshead displacement was set at 3 mm to simulate a cuff opening large enough to place the device around a nerve of 1 mm diameter. Load and extension were acquired using the corresponding software Bluehill at an acquisition frequency of 100 Hz. Load‐displacement curves were obtained to enable comparison between devices. The impact of cyclic mechanical stretching on the cuffs was investigated by optical microscopy and electrochemical measurements.

##### Statistical Analysis

All reported values are means with error bars corresponding to one standard deviation. A paired sample Student's *t*‐test was used to identify differences before and after autoclave, ex vivo and mechanical testing. A Student's *t*‐test was used to identify differences between CE sheet and CE array performance. One way ANOVA followed by Bonferroni post hoc tests were performed to determine the statistical significance when comparing different types of cuffs. Statistical significance was determined by *p*‐values < 0.05.

## Conflict of interest

The authors declare no conflict of interest.

## Author Contributions

E.A.C and C.A.R.C. contributed equally to this work. E.A.C, C.A.R.C., J.A.G., and R.A.G. contributed in conceptualization. E.A.C and C.A.R.C. contributed in device manufacturing and data collection. E.A.C and O.S contributed in conducting ex vivo tests. E.A.C contributed in data analysis. C.A.R.C. and E.A.C contributed in writing the draft manuscript. C.A.R.C., J. A.G., and R. A. G. contributed in supervising the project and editing the manuscript. All authors revised the manuscript content and approved the final version of the manuscript.

## Supporting information

Supporting Information

## Data Availability

Data available on request from the authors.
