# Supplementary material for: Stretchable, Fully Polymeric Electrode Arrays for Peripheral Nerve Stimulation
Source: Adv Sci (Weinh). 2021 Feb 5;8(8):2004033. doi: 10.1002/advs.202004033 (PMC8061359; doi:10.1002/advs.202004033)
Supplement: Supplementary file 1 — Supporting Information [file ADVS-8-2004033-s001.pdf]

© 2021 Wiley-VCH GmbH

ADVANCED SCIENCE
Open Access

Supporting Information

for *Adv. Sci.*, DOI: 10.1002/advs.202004033

Stretchable, Fully Polymeric Electrode Arrays for Peripheral Nerve Stimulation

*Estelle A. Cuttaz, Christopher A. R. Chapman, Omaer Syed, Josef A. Goding and Rylie A. Green**

Stretchable, Fully Polymeric Electrode Arrays for Peripheral Nerve Stimulation

*Estelle A. Cuttaz, Christopher A. R. Chapman, Omaer Syed, Josef A. Goding and Rylie A. Green**

E. A. Cuttaz, Dr. C. A. R. Chapman, Dr. O. Syed, Dr. J. A. Goding, Dr. R. A. Green
Department of Bioengineering, Imperial College, South Kensington, SW7 2AZ, London, UK
E-mail: rylie.green@imperial.ac.uk

Supporting Information

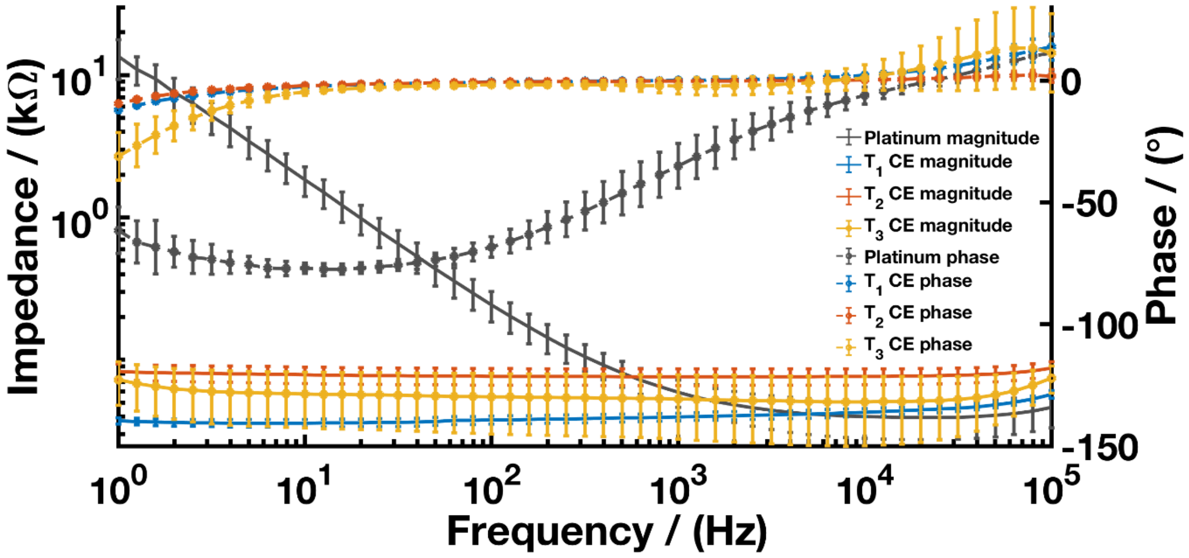

Figure S1. CE sheet performance across three thickness variants (T_1, T_2 and T_3) showing impedance magnitude in absolute values and phase angle of EIS compared to platinum. Results are reported as mean ± standard deviation (n = 5).

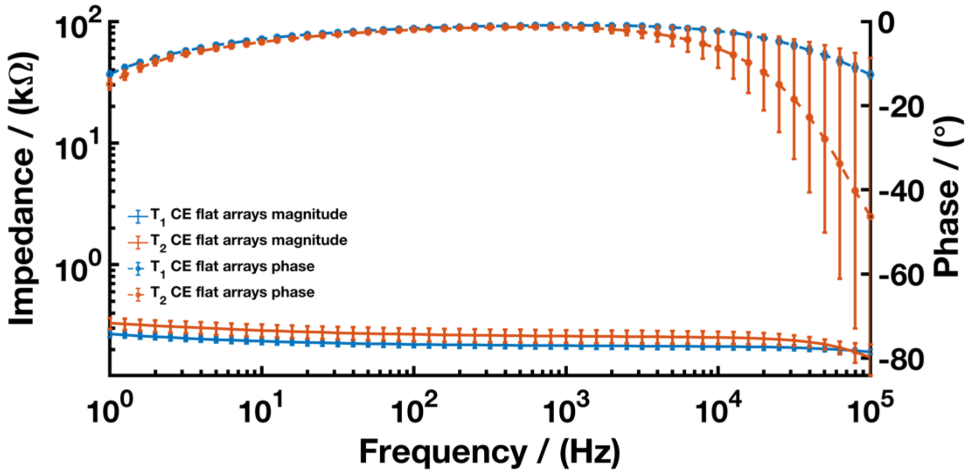

Figure S2. Impedance magnitude in absolute values phase angle of EIS comparing T_1 and T_2 CE in bulk sheet configuration and in flat array configuration. Results are reported as mean ± standard deviation (average across three different batches, n = 5 for both T_1 and T_2 CE in bulk sheet; n = 14 for T_1 CE flat arrays; n = 17 for T_2 CE flat arrays).

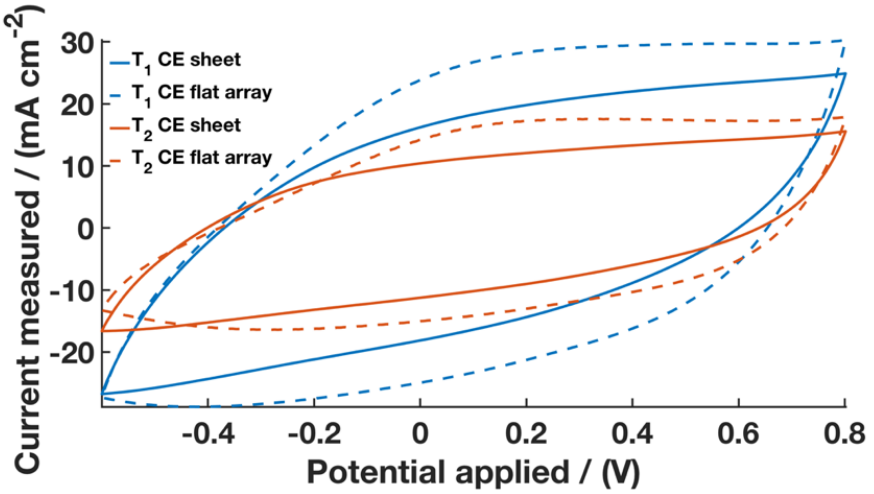

Figure S3. Cyclic voltammetry comparing T_1 and T_2 CE in bulk sheet configuration and in flat array configuration. Results are reported as mean ± standard deviation (average across three different batches, n = 5 for both T_1 and T_2 CE in bulk sheet; n = 14 for T_1 CE cuff; n = 17 for T_2 CE cuff).

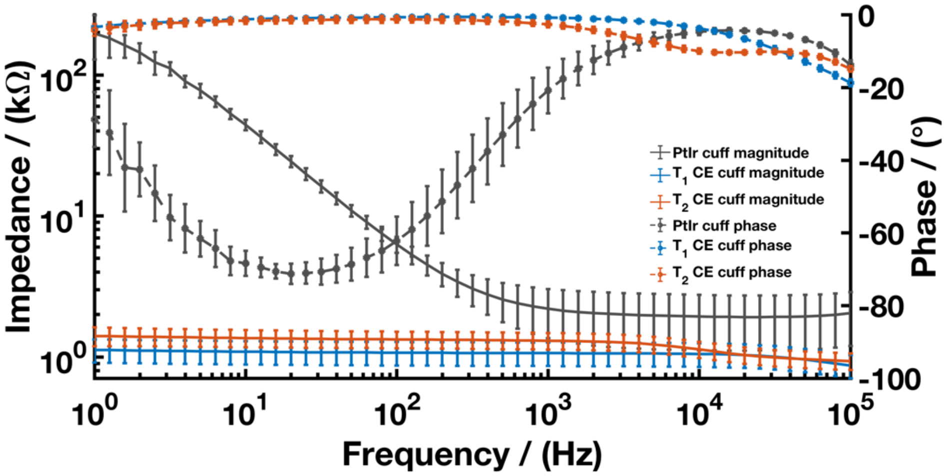

Figure S4. Impedance magnitude in absolute values and phase angle of EIS for T_1 and T_2 CE cuff arrays compared to PtIr cuff arrays. Results are reported as mean ± standard deviation (N = 12 for T_1 CE cuff, N = 15 for T_2 CE cuff).

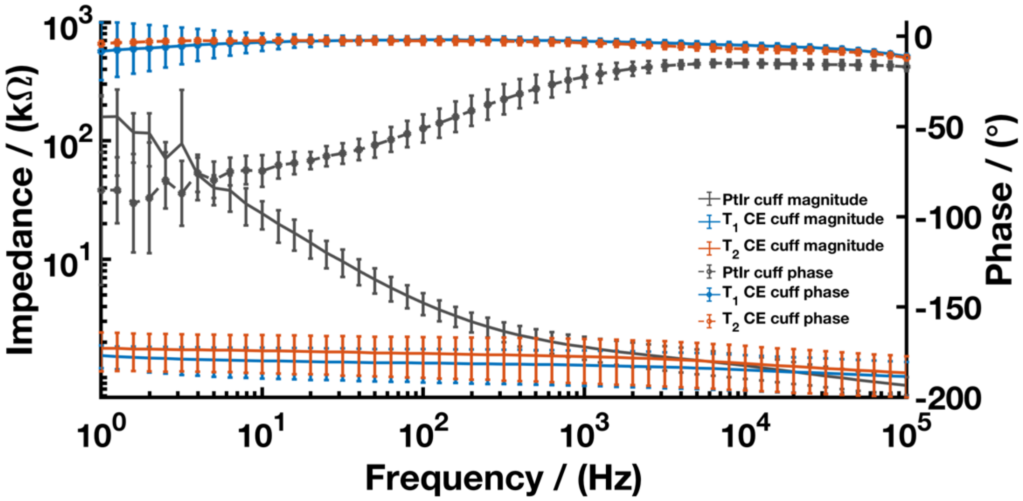

Figure S5. Impedance magnitude in absolute values and phase angle of EIS for T_1 and T_2 CE cuff arrays performance during ex vivo compared to PtIr cuff arrays. Results are reported as mean ± standard deviation (N = 11 for T_1 CE cuff, N = 15 for T_2 CE cuff).

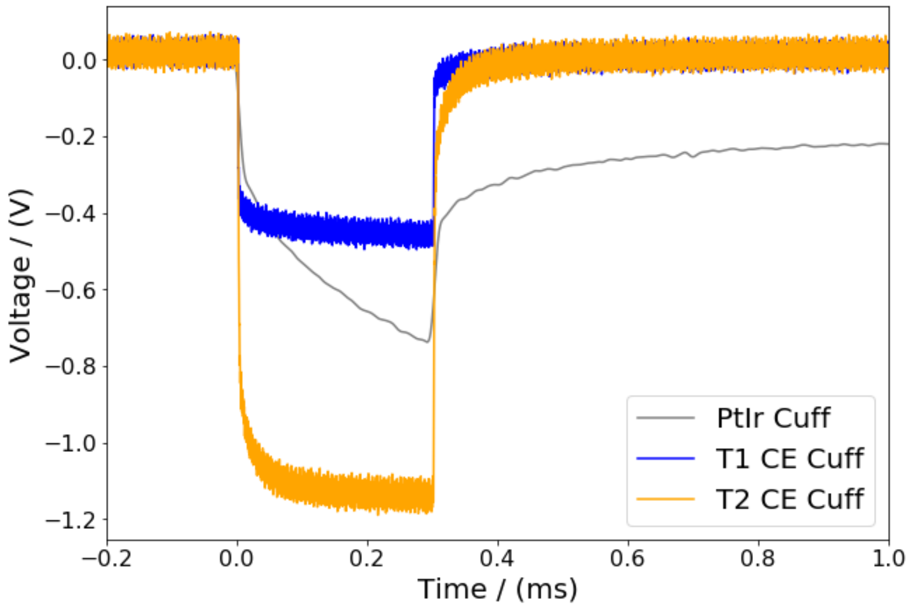

Figure S6. Representative voltage transients obtained for PtIr cuff, T_1 CE cuff and T_2 CE cuff at a pulse width of 300 µs and a current amplitude of 500 µA during *ex vivo*.

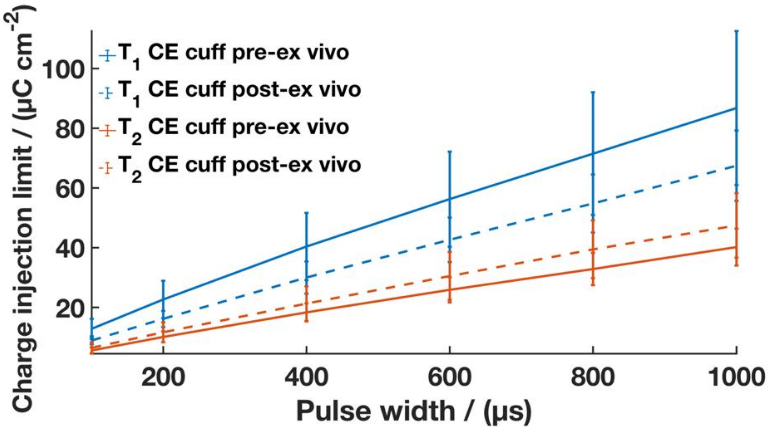

Figure S7. CIL of T_1 (solid blue line + blue dotted line) and T_2 CE cuff arrays pre (solid blue line and solid orange line) and post-ex vivo testing (dashed blue line and dashed orange line).

Table S1. Pre-and post-ex vivo values for the T_1 and T_2 CE cuffs. Results are reported as mean ± standard deviation (N = 11 for T_1 CE cuffs, N = 15 for T_2 CE cuffs, N = 3 for PtIr cuffs). A p-value < 0.05 indicates statistical significance (S).

Cuff type		Pre-ex vivo	Post-ex vivo	p-value
T_1 CE Cuff	Impedance @ 1 kHz [Ω cm^2]	23.02 ± 5.72	25.27 ± 3.00	0.06
	CSC [mC cm^{-2}]	105.05 ± 32.28	89.07 ± 13.68	0.01 (S)
	CIL @ 200 µs [µC cm^{-2}]	22.61 ± 6.26	16.13 ± 2.73	< 0.001 (S)
T_2 CE Cuff	Impedance @ 1 kHz [Ω cm^2]	36.41 ± 6.91	32.29 ± 10.02	0.003 (S)
	CSC [mC cm^{-2}]	68.82 ± 13.46	79.13 ± 20.99	< 0.001 (S)
	CIL @ 200 µs [µC cm^{-2}]	10.11 ± 1.80	11.70 ± 3.32	0.005 (S)
PtIr Cuff	Impedance @ 1 kHz [Ω cm^2]	27.28 ± 11.63	22.73 ± 2.35	0.22
	CSC [mC cm^{-2}]	2.29 ± 0.35	2.51 ± 0.55	0.25
	CIL @ 200 µs [µC cm^{-2}]	10.65 ± 1.81	9.69 ± 2.64	< 0.001 (S)

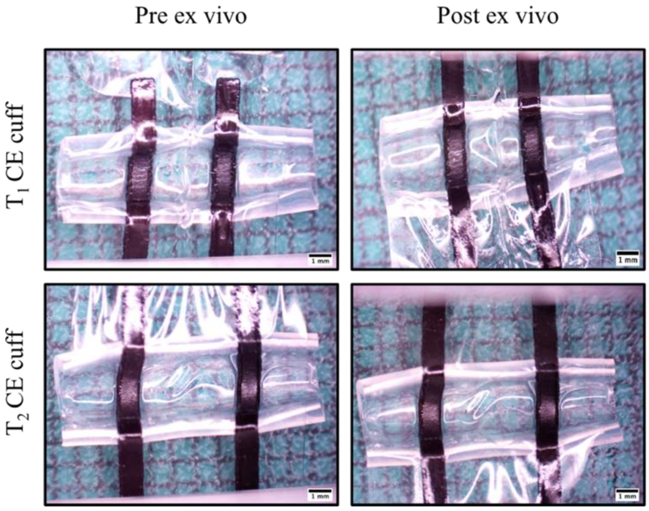

Figure S8. Stereoscope images showing a top view of T_1 and T_2 CE cuff arrays pre-and post ex vivo.

Table S2. Pre-and post-cyclic tensile testing values for the T_1, T_2 CE and PtIr cuffs. Results are reported as mean ± standard deviation (N = 5 for T_1 CE cuffs, N = 4 for T_2 CE cuffs, N = 2 for PtIr cuffs). A p-value < 0.05 indicates statistical significance (S).

Cuff type		Pre-cyclic tensile testing	Post-cyclic tensile testing	p-value
T_1 CE Cuff	Impedance @ 1 kHz [Ω cm^2]	27.61 ± 5.14	25.53 ± 4.67	0.12
	CSC [mC cm^{-2}]	86.37 ± 15.02	91.59 ± 19.83	0.27
	CIL @ 200 µs [µC cm^{-2}]	17.02 ± 3.32	17.30 ± 3.17	0.75
T_2 CE Cuff	Impedance @ 1 kHz [Ω cm^2]	40.84 ± 10.43	33.89 ± 11.10	0.006 (S)
	CSC [mC cm^{-2}]	63.71 ± 17.13	76.01 ± 18.92	0.008 (S)
	CIL @ 200 µs [µC cm^{-2}]	10.69 ± 1.43	13.76 ± 4.42	0.06
PtIr Cuff	Impedance @ 1 kHz [Ω cm^2]	22.35 ± 1.57	20.02 ± 3.86	0.31
	CSC [mC cm^{-2}]	2.89 ± 0.25	3.26 ± 0.91	0.40
	CIL @ 200 µs [µC cm^{-2}]	11.05 ± 1.60	12.66 ± 0.96	0.12

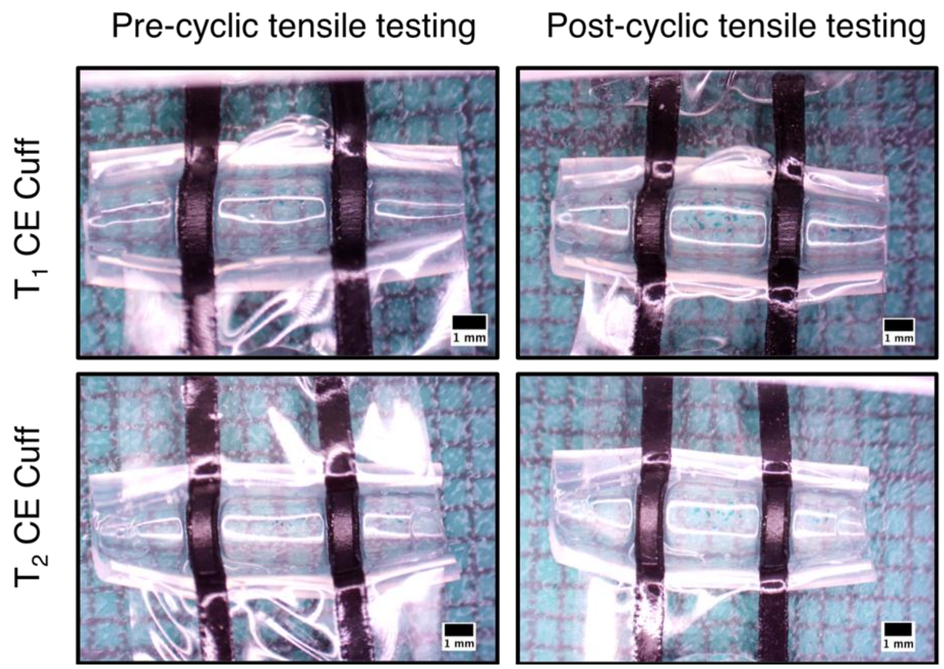

Figure S9. Stereoscope images showing a top view of T_1 and T_2 CE cuff arrays pre and post cyclic tensile testing

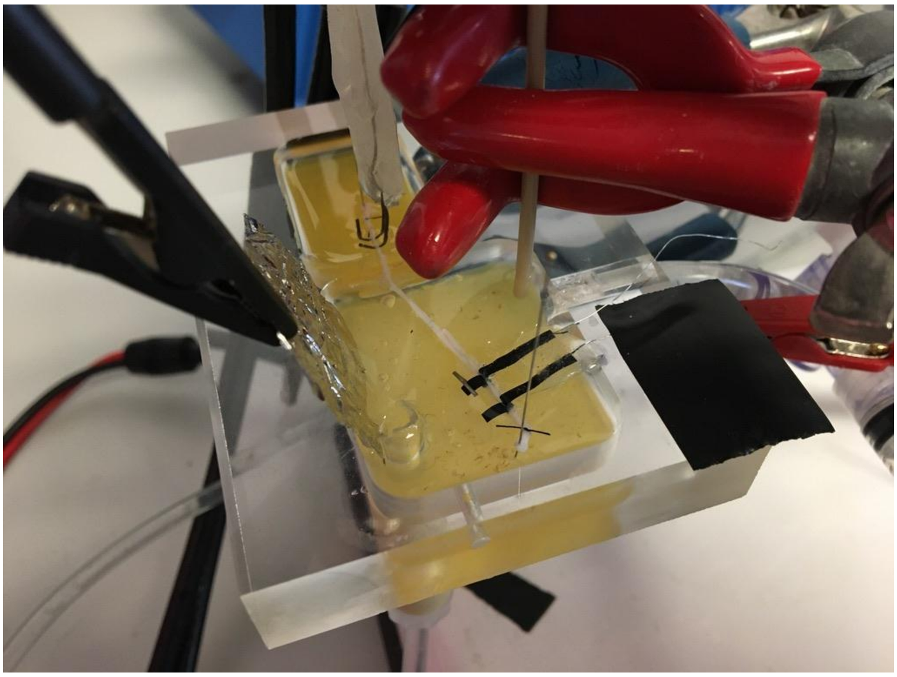

Figure S10. Picture of the *ex vivo* set up
